# Epidemiological analysis of rubella-confirmed cases from measles-suspected cases in Ethiopia: threat for congenital rubella syndrome

**DOI:** 10.1017/S0950268822000279

**Published:** 2022-02-28

**Authors:** Diriba Sufa Gemechu, Yoseph Worku, Zewdu Assefa Edae, Yohannis Dugasa Feyisa, Shambel Habebe Watere, Abyot Bekele Woyessa, Abebe Dukessa, Urge Gerema

**Affiliations:** 1 Ethiopian Public Health Institute, Addis Ababa, Ethiopia; 2 Saint Paul's Hospital Millennium Medical College, Addis Ababa, Ethiopia; 3 Department of Biomedical Sciences, Institute of Health, Jimma University, Jimma, Ethiopia

**Keywords:** Congenital, epidemiology, Ethiopia, rubella, surveillance, syndrome

## Abstract

Rubella is a highly contagious mild viral illness. It is a leading cause of congenital rubella syndrome (CRS). Routine data of rubella do not exist in Ethiopia. However, laboratory-based conformation of rubella cases from measles negative samples were collected from a measles surveillance system. The current study was to analyse the epidemiological distribution of rubella cases from measles-suspected cases in Ethiopia from 2011 to 2015. National-based secondary data analysis of rubella through measles-based surveillances was carried out. Measles-suspected cases were investigated using the case investigation form, and a serum sample collected and sent to the Ethiopian laboratory for conformation. Samples tested for measles immunoglobulin M (IgM) were tested for rubella. The investigation results were entered into an electronic database using SPSS version 25 for analysis. Out of 11749 samples tested for rubella IgM from 2011 to 2015, 2295 (19.5%) were positive for rubella IgM and 51% of rubella-positive cases were female. Five per cent of all cases were female aged between 15 and 49. Cases were confirmed from all regions, two administrative towns and seasonal variations were observed with peaks in the first and fourth seasonal periods of the years. As fear of congenital abnormality (CRS), the Ethiopian government should focus on rubella syndrome surveillance with the aim of starting a rubella vaccine.

## Introduction

Rubella is a communicable viral infection [1, 2]. It is a common infection of children and it is characterised by a skin rash. However, rubella virus results in congenital abnormalities such as hearing problems, eye problems and heart disease when pregnant women were infected by rubella virus during pregnancy [3–6]. Rubella virus is a known teratogen and it is a major public health concern, because it results in high mortality [1, 2]. Various countries have used different methods to reduce the transmission of rubella virus, such as vaccination which is cost effective and safe [3, 4]. Vaccines are available in most of the countries [5, 6].

A safe and effective immunisation is available for the prevention of rubella for those aged more than 45 years, but there is no global recommendation for inclusion of the vaccine in that given in regular-based schedule in most developing countries, including Ethiopia. The impact of the rubella infection at the national and international level is needed for different countries to make informed decisions on the inclusion of rubella vaccine in their national programme [7, 8].

The total population of Ethiopia was around 110 million according to the 2019 population projection [9], out of which only 19% of the population were living in urban areas. From a total population, about 12.5% were children less than 5 years, and 90% of the population has access to health care services [10]. On an average the estimated life expectancy was estimated to be 57 years and the total fertility rate is 5.3 [11–14]. The country has 1.1 million square kilometres of area. There are more than 80 linguistic groups in Ethiopia. Rubella and its complication, CRS, can be prevented by vaccination. Rubella vaccination is not introduced into the infant vaccination schedule in Ethiopia. Ethiopian programme on immunisation (EPI) was introduced in the 19th century. This involves administering the first dose of the measles vaccine after the 9th month after delivery [11, 15].

Ethiopia does not contain rubella CRS surveillance and information on the incidence of rubella and CRS is limited. Therefore, the analysis aimed to figure out the results of rubella testing from national measles surveillance and it provides an epidemiological explanation of the rubella cases in Ethiopia during 2011–2015.

The analyses of the epidemiology of rubella testing, in parallel with measles case-based surveillance, provided an estimate of the epidemiology of rubella disease in Ethiopia indicating the disease is endemic in the country. In addition, sentinel surveillance for CRS and sero-prevalence studies, to assist with defining a rubella susceptibility profile. These elements necessarily determine the burden of rubella in the country, identify, plan, implement and evaluate appropriate control strategies for the disease by the ministry of health, different stalk holders, local health planners and researchers.

## Methods

### Study design and population

A descriptive analysis of retrospective secondary surveillance data was collected from the Ethiopian National laboratory. All measles suspected cases, whose laboratory specimen is taken and brought to European Public Health Institute (EPHI) and all rubella tested measles suspect cases from 2011 to 2015. The study was conducted in nine regional states and two administrative towns in Ethiopia [16]. Ethiopian national measles laboratory was one of the global vaccine-preventable diseases laboratory networks and it had been accredited annually since 2004.

### Data collection and processing procedure

Measles control activities started from establishing measles surveillance in Ethiopia from 2014 in order to control the impact of measles, following a recommendation from the World Health Organization [6]. The surveillance was done by using common procedures and collecting samples from the suspected measle cases [12]. The samples were sent to an Ethiopian nationally accredited lab for measles in the EPHI for laboratory testing using a standard procedure to detect IgM [7].

The samples were taken from those cases that had not been administered measles vaccination over the past 4 weeks before the specimen was collected and categorised as cases of measles which is confirmed by the presence of immunoglobulin M (IgM). A specimen which is negative or indeterminate result of a measles case investigated for rubella immunoglobulin antibody. Cases confirmed with rubella immunoglobulin antibody positive were categorised as confirmed for rubella. The results of laboratory and individual case investigation data were entered into different databases and then data were extracted and analysed using SPSS version 25.

## Results

A total of 11 749 specimens were tested for rubella IgM from measles-suspected cases coming through measles case-based surveillance from 2011 to 2015 for rubella IgM. Of these, 2295 (19.5%) were laboratory-confirmed rubella cases, 8262 (70.3) had negative test results for rubella IgM and 1192 (10.1%) had indeterminate results. The numbers of confirmed rubella cases were 159 in 2011, 783 in 2012, 814 in 2013, 215 in 2014 and 324 in 2015 among the total specimens tested for rubella IgM in each year (Table 1).

**Table 1.** Number of specimens tested for rubella antibodies and results, Ethiopia, 2011–2015

Year	No. of specimens tested for rubella IgM	No. (%) of rubella IgM positive	No. (%) ofrubella IgM negative	No. (%) indeterminate
2011	1295	159 (12.3)	950 (73.4)	186 (14.4)
2012	3135	783 (25.0)	1933 (61.7)	419 (13.4)
2013	3242	814 (25.1)	2122 (65.5)	306 (9.4)
2014	1923	215 (11.2)	1562 (81.2)	146 (7.6)
2015	2154	324 (15.0)	1695 (78.7)	135 (6.3)
Total	11 749	2295 (19.5)	8262 (70.3)	1192 (10.1)

**Fig. 1.**
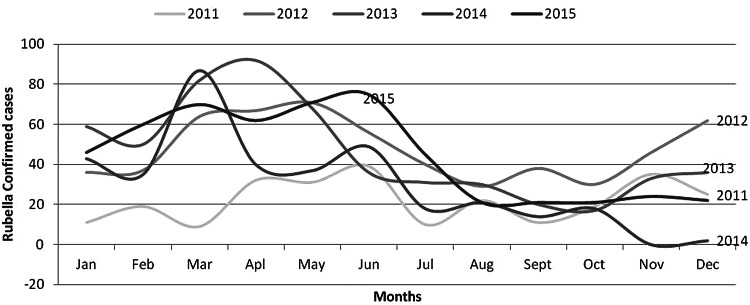
Distribution of rubella cases by months of each year, Ethiopia 2011–2015.

From the total tested specimens for rubella IgM, 5648 (48.1%) were female. About 10% and 9.5% were positive for rubella among the investigated specimens for IgM. With regards to age categories about 1310 (11.1%) were within the age group 5–14 among the total tested specimens for rubella IgM (11 749). From all confirmed cases, 51% were females aged from 2 months to 42 years with a mean age of 7 years and 6 months. About 92.1% of cases were aged less than 15 years of age, around two-third of cases (76.5%) were <10 years while 35% of cases were aged <5 years (Table 2). From 2011 to 2015, laboratory-confirmed rubella cases were identified from different regions of Ethiopia. Rubella cases were widely distributed across the country (Table 2). The distribution of rubella cases occurred each year and was high from March to June and there was observed annual seasonality of congenital rubella, with an increase in the number of cases since February. A minor peak was also observed between October and December. A high number of rubella confirmed cases was reported during 2012 (34.1%) and 2013 (35.5%). The number of confirmed cases was lower throughout 2011 relative to the other years and similarly, the number of specimens tested was also lower (Fig. 1). The number of laboratories that confirmed rubella cases was lower in 2011 and then arose to a peak in 2012 and 2013. Again it decreased in 2014.

**Table 2.** Age distribution of laboratory-confirmed rubella cases, Ethiopia 2011–2015

Age group	Total specimens tested	Rubella IgM positive	Per cent	Population (av. mid. year)	Incidence rate/ 100 000 population
<1	1008	118	1.0	21 144 776	0.6
1–4	2575	685	5.8	10 966 091	6.2
5–14	6792	1310	11.1	38 524 673	3.4
15–44	1348	182	1.5	27 236 047	0.7
≥45	26	0	0.0	–	–
Total	11 749	2295	19.5	97 871 587	2.3

## Discussion

Epidemiological analysis of rubella from suspected measle cases in Ethiopia from 2011 to 2015 indicated that rubella infection affects young children and it was widely distributed in different regions of the country. The current study is consistent with previous studies which indicate that the rubella infection was widely distributed throughout the country [17]. From 2011 to 2015, around 2295 laboratory-confirmed rubella cases were identified from 11 regions and two administrative towns of Ethiopia. Among these cases, 92% rubella infections occurred in young children aged <15 years of age which revealed that the infections were prevalent in school going children (Table 3).

**Table 3.** Distribution of laboratory-confirmed rubella cases by region, Ethiopia 2011–2015

Region	Total specimens tested for rubella	Rubella IgM positive	Positivity rate (%)	Population (av. mid population)	Incidence rate/100 000
Addis Ababa	1847	473	25.6	3 167 036	14.9
Afar	167	29	17.4	1 643 280	1.8
Amhara	2054	448	21.8	19 370 012	2.3
Benishangul	254	67	26.4	825 057	8.1
Dire Dawa	79	19	24.1	407 514	4.7
Gambella	61	25	41.0	406 607	6.1
Harari	74	11	14.9	219 431	5.0
Oromia	4909	672	13.7	33 175 153	2.0
SNNPR	1833	470	25.6	18 375 050	2.6
Somali	105	4	3.8	5 312 893	0.1
Tigray	366	77	21.0	5 128 532	1.5
Total	11 749	2295	19.5	88 030 565	2.6

According to this report, the age group from 1 to 4 was more affected, with an incidence rate of 6.4 per 10 000 populations. The current study can be compared with studies conducted across different regions of Ethiopia by using the serological method [9, 13]. From the above study, the incidences of congenital rubella sero-positivity were found to be ≥94% among individuals less than 14 years of age. This result is also similar to a study conducted in Zimbabwe by using a review of surveillance of measles secondary data in which 94% of confirmed cases were less than 15 years of age [14, 18, 19]. Routine surveillance and high priority should be given to children, especially those among age group from 1 to 4.

In the current study, the number of cases were varying from season to season, with an increase in the month of February and a decrease to the lowest level in the last month of summer [17, 20]. The burden of rubella varies from season to season peaks in the month of February to better understand establish rubella surveillance and sero-prevalence testing.

Measles case-based surveillance was employed to study rubella cases in a setting in Ethiopia. From this surveillance, rubella cases were identified, despite the absence of passive surveillance and low sensitivity. From all regions, a high amount of rubella cases was found in wider transmission [8]. The current results were comparable with previous studies in which the prevalence of congenital rubella syndrome (CRS) ranged from 0.1 to 0.2 cases per live births [19, 21, 22].

African countries including Ethiopia did not include the rubella vaccine within the EPI and the routine surveillance. From the current findings, based on the measles surveillance the results for rubella and measles antibodies were negative. This revealed that low predictive value for the diseases and case definition for rubella and CRS are pending field testing [13, 16, 23]. This may be the period during which data were collected.

However, according to a protocol of surveillance, the results of the collection of samples within the first 30 days after the onset of the rash may be missed. The standard protocol of rubella surveillance and testing procedure of rubella should be established.

### Merits of the study

The study has some advantages, this study used laboratory-based data from the participants with a huge sample size from all regions of the country which is more representative. The data were gathered from the entire health care system including private health facilities. This study also identifies the prevalence of rubella in Ethiopia which is used for early diagnosis and to design appropriate prevention strategies for the disease.

### Limitation

**Fig. 2.**
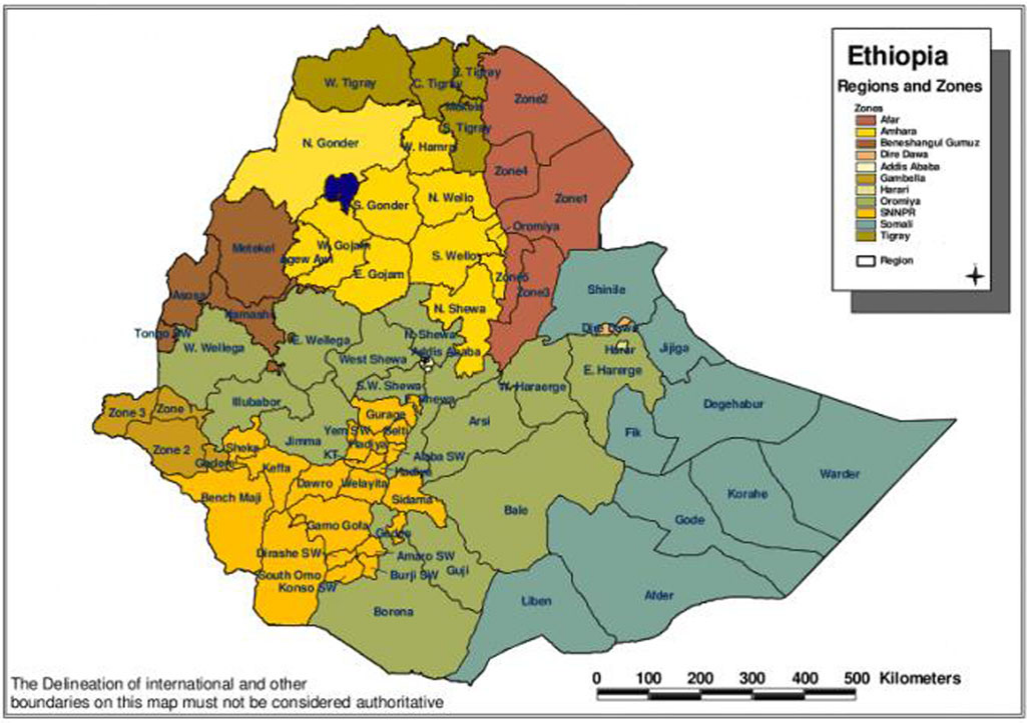
Map of Ethiopia.

Data used were secondary in nature and incompleteness and inaccuracies data were excluded. We did not have locations of these places/regions, so it was difficult to draw the map of Ethiopia to show us the distribution over time using QGIS. The objectives of the study were to identify the epidemiology of rubella, we did not conduct bivariate or logistic regressions with rubella test outcome behind variable interest (Fig. 2).

## Conclusions

Epidemiological analysis of rubella infection from suspected measle cases was carried out in Ethiopia and infection was prevalent among young children and it was widely distributed in different regions of the country.

The prevalence of rubella is increases from time to time, so health providers should routinely examine women of childbearing age for immunity and vaccinate those who lack acceptable immunity. The health provider should isolate the patient with rubella for 7 days after they develop a rash and they should be aware of the outbreak measures that should begin as as soon as the rubella outbreak is suspected. In addition, community mobilisation to create awareness on the knowledge and attitude on the rubella virus is essential. Furthermore, studies should be conducted especially on different risk factors associated with the rubella virus.

## Data Availability

Data used to analyse the present study are available with the corresponding author on reasonable request.
